# Diabetes, Obesity, and Inflammation: Impact on Clinical and Radiographic Features of Breast Cancer

**DOI:** 10.3390/ijms22052757

**Published:** 2021-03-09

**Authors:** Braden Miller, Hunter Chalfant, Alexandra Thomas, Elizabeth Wellberg, Christina Henson, Molly W. McNally, William E. Grizzle, Ajay Jain, Lacey R. McNally

**Affiliations:** 1 Department of Surgery, University of Oklahoma Health Sciences Center, Oklahoma City, OK 73104, USA; Braden-Miller@ouhsc.edu (B.M.); Hunter-Chalfant@ouhsc.edu (H.C.); 2 Department of Internal Medicine, Wake Forest University School of Medicine, Wake Forest University, Winston-Salem, NC 27157, USA; althomas@wakehealth.edu; 3 Department of Pathology, University of Oklahoma Health Sciences Center, Oklahoma City, OK 73105, USA; elizabeth-wellberg@ouhsc.edu; 4 Department of Radiation Oncology, University of Oklahoma Health Sciences Center, Oklahoma City, OK 73105, USA; christina-henson@ouhsc.edu; 5 Stephenson Cancer Center, Oklahoma City, OK 73104, USA; mwmcnally@hotmail.com; 6 Department of Pathology, University of Alabama at Birmingham, Birmingham, AL 35294, USA; wgrizzle2@gmail.com

**Keywords:** diabetes, obesity, imaging, molecular imaging, breast cancer

## Abstract

Obesity, diabetes, and inflammation increase the risk of breast cancer, the most common malignancy in women. One of the mainstays of breast cancer treatment and improving outcomes is early detection through imaging-based screening. There may be a role for individualized imaging strategies for patients with certain co-morbidities. Herein, we review the literature regarding the accuracy of conventional imaging modalities in obese and diabetic women, the potential role of anti-inflammatory agents to improve detection, and the novel molecular imaging techniques that may have a role for breast cancer screening in these patients. We demonstrate that with conventional imaging modalities, increased sensitivity often comes with a loss of specificity, resulting in unnecessary biopsies and overtreatment. Obese women have body size limitations that impair image quality, and diabetes increases the risk for dense breast tis-sue. Increased density is known to obscure the diagnosis of cancer on routine screening mammography. Novel molecu-lar imaging agents with targets such as estrogen receptor, human epidermal growth factor receptor 2 (HER2), pyrimi-dine analogues, and ligand-targeted receptor probes, among others, have potential to reduce false positive results. They can also improve detection rates with increased resolution and inform therapeutic decision making. These emerg-ing imaging techniques promise to improve breast cancer diagnosis in obese patients with diabetes who have dense breasts, but more work is needed to validate their clinical application.

## 1. Introduction

Inflammation is a hallmark of cancer [1] and is associated with its development and progression, though this relationship is quite complex and not fully understood [2]. While chronic inflammation or infections can promote cancer development, the immune system also has a large role in surveillance and killing tumors as they arise [3]. Breast cancer, the most common malignancy in women in the United States [4,5,6], has been associated with inflammation [7]. This association is not as direct, however, as other solid tumors such as hepatocellular cancer that can result from chronic inflammation associated with hepatitis B and C infection [8]. The recently published Women’s Health Initiative trial, which prospectively enrolled over 27,071 participants with a median follow-up of 19 years, attempted to better evaluate the relationship between inflammation and breast cancer pathogenesis [9]. Specifically, it correlated the levels of four circulating inflammatory biomarkers (high-sensitivity C-reactive protein, fibrinogen, N-acetyl side-chains of acute phase proteins, and soluble intercellular adhesion molecule-1) with breast cancer risk. The data suggest that there is indeed a correlation, as elevated fibrinogen was directly associated with increased breast cancer incidence. Soluble intercellular adhesion molecule-1 had an inverse association, and the other two biomarkers had no association with the development of breast cancer [9]. These data underscore the complexity of the intersection of inflammation and breast cancer, but also suggest that management of inflammation may have a role in prevention.

It is possible that other common, chronic diseases increase inflammation and breast cancer risk, and therefore all patients should not be screened using a one-size-fits-all approach. Specifically, diabetes and obesity have been associated with chronic inflammation and breast cancer [10,11]. Both disorders are increasing in prevalence, and the age of diagnosis for each is decreasing [12]. Similarly, the annual incidence of breast cancer has been increasing, albeit at a slower rate (0.3% per year) (American Cancer Society Facts and Figures) [13]. A better understanding of the relationship between obesity, diabetes, inflammation, and breast cancer could have important implications for multiple aspects of breast cancer management, including prevention, diagnosis, and treatment.

While a full evaluation of the complex interplay between obesity, diabetes, inflammation, and breast cancer is beyond the scope of this review, we wish to explore whether these disorders negatively impact our ability to diagnose breast cancer and whether alternative diagnostic strategies may better serve patients with these chronic diseases. The diagnosis of ductal carcinoma in situ (DCIS) remains controversial and is not covered in this review. Given that early detection of breast cancer through screening mammography has been a mainstay for reducing breast cancer-specific mortality, we specifically focus on whether obesity, diabetes, and inflammation negatively impact the efficacy of standard contemporary breast imaging platforms [13]. Furthermore, we explore novel molecular imaging agents and techniques that may increase the sensitivity and specificity of breast cancer diagnostics in patients with these disorders. Lastly, we discuss the potential role for anti-inflammatory agents in improving our ability to detect breast cancer through standard and novel imaging approaches.

## 2. Body Sections

### 2.1. Obesity-Related Limitations to Conventional Imaging

There are numerous data to support that obesity is associated with increased breast cancer risk and disease severity in postmenopausal women [10]. One meta-analysis reported that the risk ratio for breast cancer was 1.12 (95% CI, 1.08–1.16) for each 5 kg/m^2^ increase in BMI [14]. Obese women also have worse breast cancer-specific survival [15]. Clinical diagnosis can be challenging in the obese population as primary lesions and enlarged lymph nodes are less palpable, contributing to lower rates of symptom-detected breast cancers [16,17]. Given the increased time required for clinical symptoms to develop in the obese population, imaging-based screening techniques play a more important role in diagnosis [18]. Although there is evidence that obese women are less likely to undergo routine screening mammography, this does not account for increased rate of breast cancer in these patients. Prospective data have shown higher rates of advanced cancer in women with higher BMI, regardless of the extent of mammography use with equal false-negative rates compared to normal or underweight women [19]. The data suggest that despite decreased rates of mammography use, obesity represents an independent risk factor for breast cancer development [10,19,20].

**Figure 1:**
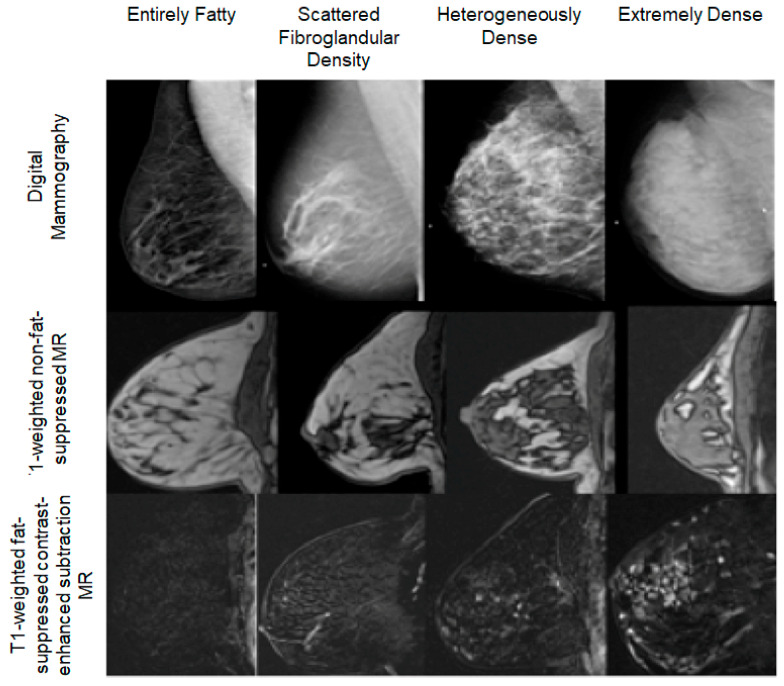
Imaging appearance of different breasts of increasing density using three types of imaging modalities. Digital mammography depicts how breasts appear more opaque as breast density increases. T1-weighted non-fat-suppressed MR images show increasing amount of fibroglandular tissue (FGT). T1-weighted fat-suppressed contrast-enhanced subtraction MR images show increasing amounts of background parenchymal enhancement (BPE), which reflects the vascularity of the fibroglandular tissue. (Adapted from [23]).

Although obesity may increase breast cancer risk, at first glance it would seem that obesity might make breast cancer easier to detect using conventional breast imaging techniques. Obese women are more likely than normal or underweight women to have fatty breast tissue, which is associated with higher mammographic sensitivity [21]. Adipose tissue serves as a radiolucent backdrop against which radiodense cancers are more easily detected. Given the known impact of breast density on sensitivity of mammography for breast cancer screening, the American College of Radiology has developed a breast density classification system based on the relative amount of fibroglandular tissue (dense) and fatty tissue (less dense), stratifying breast tissue into four groups: (A) entirely fatty; (B) scattered fibroglandular density; (C) heterogeneously dense; or (D) extremely dense [22]. The classification system was developed to help inform the provider the relative possibility that a lesion might be obscured by normal tissue. The denser the breast, the larger the lesion it may mask. Category C may obscure small noncalcified lesions whereas category D may obscure larger lesions. Categories A, B, C, and D make up 10%, 40%, 40% and 10% of the population, respectively [20]. The breast composition categories are distinct from the BI-RADS assessment categories for further work-up of suspicious findings [22]. These categories support the assertion that cancer detection should be easier in patients with obesity (Figure 1).

Since higher BMI is directly associated with more fatty breast tissue, obese women are found to have a 3% to 38% increase in sensitivity in breast cancer detection compared to normal weight women [21]. Most data suggest that increased sensitivity in fatty breast tissue is not associated with a significant loss of specificity [21,24,25], however, there are conflicting data. Other studies suggest increased BMI may be an independent risk factor for higher false positive rates after adjusting for age and breast density, with similar sensitivity [25]. The use of digital breast tomosynthesis (DBT) combined with full-field digital mammography can increase cancer de-tection rates compared to conventional mammography alone (from 4.2 to 5.4 cancers detected per 1000 examina-tions) and modestly decrease false-positive rates (15% reduction from 61.1 to 53.1 per 1000 examinations). It can also reduce recall rates, which is the percentage of mammograms that require follow-up imaging or biopsy (17–37% reduc-tion) [26,27,28,29,30]. Therefore, although the biopsy rate was higher in women who also underwent DBT (19.3 vs. 18.1 per 1000 cases), the positive predictive value of biopsy increased 21% due to greater accuracy [26]. When stratifying by breast density, it was shown that DBT did not increase detection rates in women with almost entirely fatty breasts, but the reduction in recall rates remained [31]. Despite the emergence of DBT, there continues to be a need to decrease false-positive results to reduce the financial and psychological cost of unnecessary clinical testing.

The addition of magnetic resonance imaging and ultrasound, on the other hand, improves cancer detection rates but fails to reduce unnecessary biopsies [32,33]. Magnetic resonance imaging has higher sensitivity than mammography or ultrasound (84% vs. 39% vs. 39%, respectively), and it is often used as a first line screening tool in women who are deemed to be at high risk for breast cancer (20% lifetime risk or greater) [34]. The sensitivity of MRI in detecting contralateral lesions is between 88–100% versus 19–56% for conventional imaging [35,36], however, preoperative MRI to investigate the extent of disease is controversial. The data consistently suggest that the use of preoperative MRI leads to increased rates of more radical surgery without evidence that it improves overall survival or reduces need for re-operation [37]. Rates of mastectomy are 1.5–2 times higher in patients who receive preoperative MRI versus those who did not [38,39]. The drawbacks to MRI include high false-positive rate, high cost, and increased time to surgery to biopsy suspicious lesions. There are patients who benefit from appropriate conversion to more extensive surgery, and there is a need to identify these patients with higher fidelity [40]. The risk of overtreatment or unnecessary biopsy due to inaccurate imaging modalities may be mitigated by novel imaging techniques with equal or superior sensitivity.

**Figure 2:**
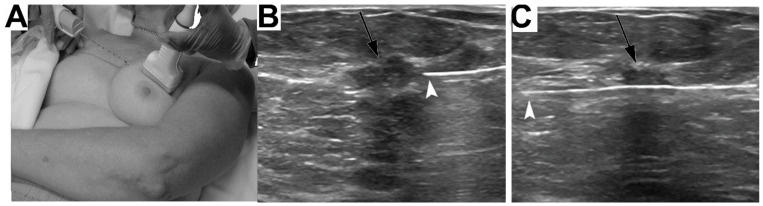
Image of ultrasound placement for ultrasound-guided biopsy. (**A**) Image of ultrasound-guided biopsy for medial breast lesions. (**B**) In the prefire position, needle tip (white arrowhead) should be placed at the margin of most lesions (black arrow), with needle parallel or near parallel to chest wall. (**C**) Postfire images should be documented and should definitively outline the needle traversing the lesion (black arrow). The tip of the needle may lie beyond the lesion margin in some cases, depending on the size of the target (white arrowhead).

Besides the concerns with specificity and sensitivity, there is a host of technical challenges specific to obese patients that limit the appropriate use of conventional screening modalities [41]. Increased tissue thickness and fatty attenuation of the ultrasound waves can diminish ultrasound-guided image quality (Figure 2). There are table weight and aperture restrictions for CT and MRI scanners may also limit access for morbidly obese patients, with some larger scanners being available only in select institutions. Similarly with mammography, there are plate size limitations that do no accommodate large breasts effectively, in some cases requiring the transposition of multiple images and difficulty for the technologist to capture the nipple in profile without sacrificing the image quality of the posterior breast tissue [41]. Mammography of fatty breast tissue also leads to compressed breast thickness, especially in the mediolateral oblique view, which distorts the image quality by decreasing contrast resolution and sharpness [42,43]. Longer exposure times are required for large breasts and this increases the risk of motion artifact, which can be minimized by increasing the peak kilovoltage at the expense of image contrast [41]. In summation, there is a substantial body of evidence describing the technical limitations inherent to imaging obese patients relating to tissue composition, imaging equipment, and proper tissue positioning.

### 2.2. Diabetes-Related Changes in Imaging Quality

Although diabetes is associated with obesity, it is also an independent risk factor for breast cancer development. As many as 18% of patients with breast cancer carry a diagnosis of diabetes, which is significantly higher than the estimated percentage of the US population with diabetes in 2018 (10.5%) [11,44,45]. Even so, the underlying mechanism for this association is not fully understood. Meta-analyses suggest that diabetic women have a 20–25% increased risk of breast cancer and a 24–44% increased risk of breast cancer mortality [11,46,47]. Although diabetes and breast cancer also both share obesity as a common risk factor, the association between diabetes and increased breast cancer risk remains even after controlling for obesity [47]. Diabetes-related factors like hyperglycemia, hyperinsulinemia, and chronic inflammation have been found to facilitate tumorigenesis [47,48]. While insulin receptor signaling itself has been associated with tumor development, the primary mechanism for the proliferative effects of hyperinsulinemia are thought to be mediated by insulin-like growth factor 1 (IGF-1) receptor signaling [48,49]. In addition to increased levels of circulating IGF-1, insulin and IGF-1 share ~50% homology, leading to cross talk with the IGF-1 receptor and disrupting the phosphatidylinositol 3-kinase (PI3K)/protein kinase B (Akt) and mitogen-activated protein kinase (MAPK) pathways [48,50,51]. With the incidence of diabetes increasing throughout the world, it is becoming increasingly important to understand the impact of diabetes on cancer diagnosis.

Diabetes has been associated with increased breast density. If diabetes is truly a “driver” of breast density, data suggest it may actually both directly increase breast cancer risk and impair detection. Mammographic density is an independent predictor of breast cancer risk, and it has been studied as an intermediate phenotype to identify factors related to breast cancer etiology [52,53]. In one well-designed study of density using mammography screening data and a fully automated volumetric method of measuring and categorizing density, insulin-treated women with type 1 or type 2 diabetes were found to have greater percent dense and absolute dense volumes compared to age-matched women without diabetes [54]. The increased breast density was associated with long-term insulin use (>5 years), and women with diabetes treated with non-insulin agents had lower density [54]. Furthermore, patients with insulin resistance or elevated plasma IGF1 are more likely to have dense breast tissue [55,56,57]. There are other studies, however, that show an insulin-independent association with diabetes and breast density [58]. Although there are data linking diabetes to increased breast density, the screening accuracy in this population has not been studied as thoroughly as in the obese population.

Increased breast density in diabetic patients also complicates cancer screening and diagnosis. Dense breast tissue on mammography can resemble breast cancer, and its opacity obscures the visualization of pathologic lesions through what has been termed the “masking effect” [59]. In dense breasts, there is an increased likelihood of false positive and false negative findings [60]. The sensitivity of screening mammography in asymptomatic women with dense breasts was found to be 48% compared to 78% for the entire cohort of patients with the full range of breast density [61]. The use of DBT was found to lower recall rates and increase detection rates for women with dense breasts when comparing BI-RADs categories A and B to C and D [62,63]. When evaluating the highest density category alone (BI-RADS category D), however, there was no advantage to using DBT compared to digital mammograph [31,63]. The 10% of women with extremely dense breasts do not benefit from DBT and represent a population that may benefit most from novel imaging modalities.

**Figure 3:**
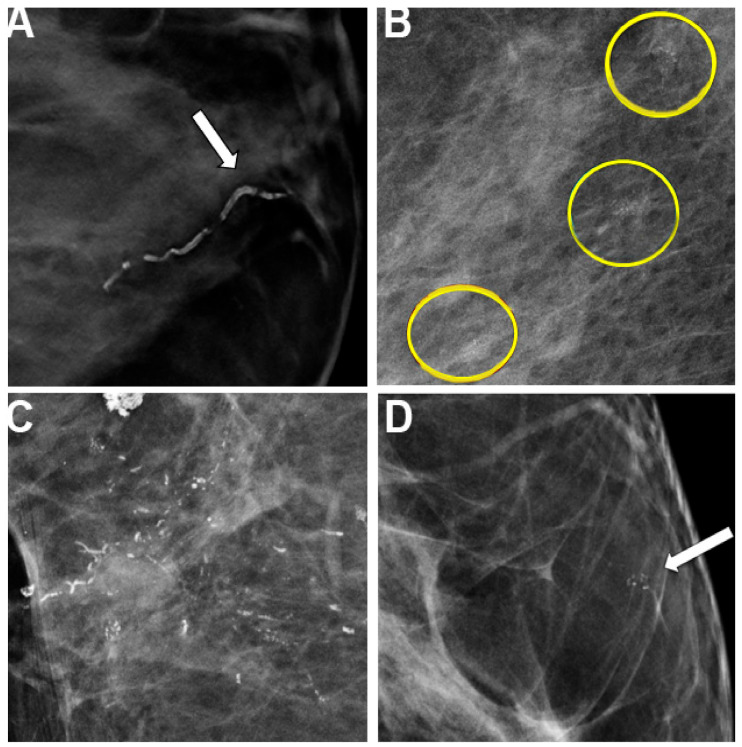
Mammography images depicting different appearances of calcifications. (**A**) Classic appearance of breast arterial calcifications (BAC) (white arrow) that are easy to discriminate and associated with diabetes. (**B**) Groups of microcalcifications (yellow circles) in a woman with ductal carcinoma in situ (DCIS) is shown in its most typical appearance on digital breast tomosynthesis (DBT). (**C**) A patient with DCIS with mammographic findings showing segmental fine pleomorphic and fine-linear branching calcifications. (**D**) A skin calcification resembles microcalcifications (white arrow) and often requires alternative views to localize to the skin. Any or all of these findings can be seen in a single mammogram and can create a convoluted picture for radiologists. (Adapted from [65]).

Patients with diabetes also are at higher risk for microcalcifications. These are deposits of calcium oxalate and cal-cium phosphate within the breast tissue that appear as white specks on a mammogram that may or may not be as-sociated with malignancy [64]. Microcalcifications are present in approximately 55% of nonpalpable breast malig-nancies and are responsible for the detection of 85–95% of cases of DCIS. They can also be present in invasive can-cers (Figure 3).

Other conventional imaging methods besides mammography have been reasonably successful at overcoming the challenges posed by increased breast density. While ultrasound alone is not a useful tool to detect breast cancer because it has very low specificity, the combination of ultrasound and mammography in patients with dense breast tissue detected 27% additional cancers compared to mammography alone [66]. A recent meta-analysis found that the addition of ultrasound increased sensitivity from 74% to 96%, with a significant decrease in specificity from 93% to 87% [67]. The use of MRI for patients with dense breasts resulted in a sensitivity of 95.7% versus 39.1% with DBT [68]. Unfortunately, MRI resulted in significantly more false-positive findings, and the positive predictive value for MRI was only 19.6% versus 31.0% for DBT [68,69]. Unlike the studies on DBT, there are no data discriminating the utility of supplemental MRI or ultrasound on women with the highest breast density category. In a systematic review of combination screening for the U.S. Preventive Services Task Force, the authors concluded that additional screening with conventional imaging will detect more cancers at the expense of unnecessary biopsies for false-positive findings [69].

### 2.3. Influences of Inflammation and Anti-Inflammatory Agents in Breast Imaging

As previously stated, the chronic, low-grade inflammation associated with obesity and diabetes has been implicated in tumorigenesis through a number of mechanisms involving tissue remodeling and adipokine signaling. Increased pro-inflammatory cytokines including PGE2, TNFa, and class 1 cytokines create a local microenvironment suitable for cancer growth [48]. The influence of obesity, diabetes, hypoxia, and inflammation on breast stromal tissue has been the subject of recent study and is summarized in the reviews by Simpson et al. and Simone et al. [70,71]. The inflammatory microenvironment created by adipocytes as well as tumor cells increases aromatase expression in surrounding mesenchymal fibroblasts which may in turn drive breast cancer growth [70,72,73]. Aromatase expression increases with proximity to a tumor and also is associated with areas of increased breast density [74,75]. Bulun et al. found that tumors are more likely to be present in regions with high stromal cell-to-adipocyte ratio—i.e., regions with high aromatase expression—but also that the aromatase expression was highest in the tumor and surrounding breast tissue [74]. Thus it is hypothesized that tumors are prone to develop in regions of dense stromal tissue where aromatase levels are highest, but also that tumors contribute to the observed aromatase levels. Increased aromatase expression, and therefore elevated local estrogen production, in dense stromal regions is one explanation for the relationship between increased breast cancer risk and increased mammographic breast density [70]. This is a very reasonable hypothesis, given the well-established role of aromatase inhibitors in breast cancer treatment.

The interplay between inflammation, dense stromal tissue, and local aromatase expression also suggests that there may be a role for non-steroidal anti-inflammatory drugs (NSAIDs) in improving our ability to diagnose breast cancer in diabetic women. Several studies have evaluated various methods to inhibit aromatase expression [76,77] and there is preclinical evidence that PGE2 stimulates aromatase expression [78,79,80]. Studies evaluating inflamed breast tissue in obese women found increased levels of COX-2-derived PGE2 resulting in increased aromatase expression, compared to normal weight women [73,81]. Moreover, tumor factors also lead to increased aromatase expression. COX-2 is overexpressed by almost half of breast tumors and is correlated with worse outcomes [70,82,83]. COX-2-derived prostaglandin expression drives increased aromatase activity which is associated with dense breast areas. COX-2 drives prostaglandin expression, resulting in local aromatase activity and estrogen production, which may increase mammographic density. COX-2 inhibition with NSAIDs may therefore be a viable therapeutic strategy to reduce breast density in diabetic women [84]. This serves as the rationale for studying the utility of NSAIDs for breast cancer prevention and treatment. NSAIDs have been shown to moderately reduce breast cancer risk and possibly reduce mammographic density (another independent risk factor for breast cancer). Aspirin and other NSAIDs are inexpensive and widely available, increasing their appeal and utility as a cancer therapeutic [83,85]. A 2019 meta-analysis showed that NSAID use reduced invasive breast cancer by about 20%, with strongest effect from aspirin and COX-2 inhibitors [86].

Despite their appeal, the data on whether NSAIDs may also reduce mammographic density and improve screening accuracy remains unclear. One study using records from over 29,000 women did not reveal a significant reduction in mammographic breast density over an interval period of approximately 18 months between mammograms in cohorts of patients stratified by NSAID use [84]. They did, however, find that initiation or continuation of NSAID use was associated with an 11% to 40% increase in maintenance of low breast density in patients with low density at the outset [84].The authors did note that the study interval was relatively short, and they posited that longer-term use of NSAIDs and longer follow up could potentially reveal reduction in density [84]. Given the significant impact that breast density has on imaging sensitivity, future studies evaluating whether long-term use of NSAIDs for select patients with factors such as obesity, diabetes and dense breasts to improve screening strategies are warranted.

### 2.4. Novel Molecular Agents and Imaging Techniques to Detect Breast Cancer in Patients with Diabetes, Obesity, and Inflammation

In addition to employing strategies to improve conventional imaging accuracy, novel molecular imaging techniques and an expanding list of potential therapeutic targets promise new opportunities in the landscape of breast cancer diagnosis and surveillance. These are particularly promising for patients with obesity, diabetes, and inflammation. Molecular imaging allows for the functional assessment and measurement of molecular and cellular processes in vivo. Conventional, widely utilized molecular imaging modalities include positron emission tomography (PET), single-photon emission computed tomography (SPECT), scintimammography and optical imaging using a fluorescent dye [87]. To date, PET is the most commonly used modality, which detects annihilation photons emitted from positron-emitting radioisotopes. Indeed, molecular imaging with novel agents has the potential to inform proper drug selection based on quantified target expression, but it may also serve as an important diagnostic tool in select patient populations with limitations to conventional imaging.

The agents currently used in molecular imaging may not sufficiently overcome the issues with specificity present in the obese and diabetic population because the tracer molecules are not inherently tumor-specific. The most studied and well understood tracer for PET imaging is 18F-fluorodeoxyglucose (FDG). FDG is an analogue of glucose that does not proceed through the entire Krebs cycle in cancer tissues due to comparatively low levels of glucose-6-phosphatase, leading to its accumulation in tumors in a process called “metabolic trapping” [88]. It is preferentially taken up by more metabolically active cancer cells, but also in infected, inflamed, and other metabolically active tissues. It represents an important clinical tool for staging, assessing treatment response, and tumor recurrence, especially in asymptomatic patients who may have elevated tumor markers or equivocal conventional imaging findings. Scintimammography is another non-tumor-specific technique, which typically uses the radiopharmaceutical technetium-99m-methoxyisobutylisonitrile (^99m^Tc-MIBI) to localize breast cancer cells due to increased blood flow, mitochondria, and cell membrane hyperpolarization [89]. Both techniques are unable to reliably detect small, subcentimeter lesions in the breast or axilla. However, the addition of SPECT/CT to lymphoscintigraphy improved sentinel node identification in overweight patients with breast cancer [90]. The results of ^18^F-FDG are limited with regard to imaging the brain, urinary system, inflamed tissue, and areas of recent surgical intervention [91,92] which can show physiologically high levels of tracer uptake. Positron emission mammography (PEM) is a method that creates a high-resolution 3-D view of the breast using two parallel photon detectors to compress the breast. The sensitivity and specificity of PEM was found to be 100% and 84.5% versus 97% and 95%, respectively for whole-body PET/CT scanning [93,94]. Compared with MRI, PEM has comparable sensitivity but greater specificity, able to detect lesions as small as 3 mm in diameter [95,96].

**Figure 4:**
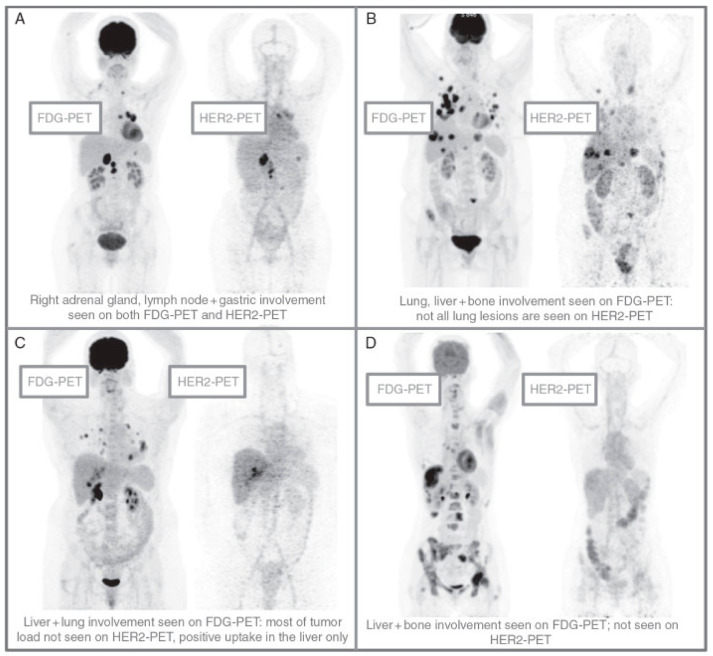
Patterns of HER2–PET/CT compared with FDG–PET/CT, Maximum intensity projection. Lesion uptake was considered concerning for malignant disease when visually higher than blood pool. (**A**) Entire tumor burden showed concerning tracer uptake; (**B**) Major portion of the tumor burden showed concerning tracer uptake; (**C**) A very small subset of the tumor burden showed concerning tracer uptake; (**D**) The entire visualized tumor burden lacked concerning tracer uptake. Adapted from [98].

The development of tracers that target cancer cells more specifically is a potential solution to imaging patients with obesity and diabetes who have dense breasts. While Human epidermal growth factor receptor 2 (HER2) is associated with aggressive disease and is overexpressed in 30% of invasive breast cancer [5,6,97], HER2^+^ breast cancer is associated with higher early survival [5]. Thus, HER2 represents a unique opportunity for monitoring of patient response using molecular imaging. Radiolabeled trastuzumab and pertuzumab (antibodies both currently used for breast cancer treatment) may help establish secondary lesions following molecular staining of biopsy or surgical specimen. This may eliminate the need for biopsy, which is especially useful in patients who refuse biopsy or when lesions are inaccessible or challenging to biopsy. At a minimum, it will reduce the risk of false negative tests and unnecessary biopsies. HER2 targeted PET tracers, i.e., HER2-PET, also have shown greater accuracy in identifying HER2 positive metastases in HER2 positive patients compared to FDG-PET (Figure 4). HER2-PET/CT accurately predicted morphological response (PPV and NPV: 100%) and discriminated patients with a median TTF of only 2.8 months (*n* = 12, 95% confidence interval) from those with a TTF of 15 months (*n* = 25, 95% confidence interval) [98].

This strategy of rescreening women with personalized tracers is highly feasible, especially with the development of nanoparticles that can more selectively deliver radiolabeled tracers to tumor cells [99]. Estrogen receptor tracers such as ^18^F-fluoroestradiol (^18^F-FES) are another tool that can be used for cancer staging, as well as to assess tumor heterogeneity and the responsiveness to endocrine therapy. Estrogen receptor tracers can also be used in combination with other classes of tracers to identify patients who require more aggressive management. A third tracer, 3′-deoxy-3′-^18^F-fluorothymidine (^18^F-FLT), a marker of proliferation, may overcome challenges to RECIST criteria assessment of treatment response. RECIST criteria measure response by quantifying the change in diameter of target lesions over time. Changes in tumor diameter, however, may not accurately reflect tumor death. Furthermore, treatment response can be seen as early as 1 week with ^18^F-FLT-PET [100], while RECIST response may require 1–2 months.

**Figure 5:**
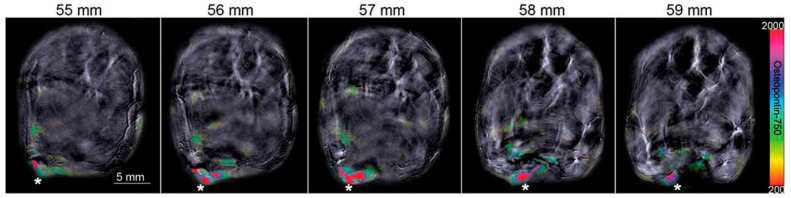
Osteopontin-probe identifies triple-negative breast cancer using multispectral optoacoustic tomography in a murine model. Serial slices are shown with * indicating positive osteopontin-probe uptake within the orthotopic triple negative breast tumor. Adapted from [103].

Many radiopharmaceutical agents are still in the investigational phase of development, and the expanding list of targets may prove useful in overcoming the heterogeneity of breast cancer on a personalized basis, particularly in triple negative breast cancers [101]. Extracellular molecules involved in cancer pathogenesis and survival are actively being investigated as potential tracers. The use of alternatively targeted imaging agents to identify triple negative breast cancer may be promising, as EGFR overexpression is seen in 57% of triple negative breast cancers. This is leading to a investigations into cetuximab treat-ment and labeled cetuximab as a molecular imaging tracer [102]. Additional targets with potential to identify triple negative breast cancer include inulin-like growth factor-1 receptor (IGF-1R), platelet-derived growth factor receptor β (PDGF- β), vascular endothelial growth factor receptor (VEGF-R), osteopontin (Figure 5), αVβ3 integrin, and claudin [96,103,104,105]. These investigational efforts are just a few examples of the promise that molecular imaging has as an adjunct to conventional anatomic imaging.

## 3. Conclusions

There is a growing body of evidence demonstrating that obesity and diabetes negatively impact breast cancer diagnosis and outcomes. Given the increased incidence of diabetes and obesity in the United States, it is important to understand how these factors influence screening results, especially since obesity and diabetes are modifiable risk factors. Despite efforts to improve conventional imaging modalities, including the use of anti-inflammatory agents to reduce breast density in order to make cancers easier to detect, the prevalence of false positive results persists. Obese women have increased sensitivity on screening mammogram, but their body habitus presents technical limitations to high quality studies. Diabetic women are more likely to have dense breasts, which have a tendency to obscure lesions through the “masking effect”. Novel image-based techniques may be useful diagnostic modalities for select populations with diabetes and obesity, but their exact role in clinical practice has yet to be elucidated. Future research is still needed to inform the creation of tailored surveillance and screening programs based on individual risk profiles.
